# Waterborne Eco-Sustainable Sol–Gel Coatings Based on Phytic Acid Intercalated Graphene Oxide for Corrosion Protection of Metallic Surfaces

**DOI:** 10.3390/ijms231912021

**Published:** 2022-10-10

**Authors:** Silvia Sfameni, Anna Del Tedesco, Giulia Rando, Fulvio Truant, Annamaria Visco, Maria Rosaria Plutino

**Affiliations:** 1 Department of Engineering, University of Messina, Contrada di Dio, S. Agata, 98166 Messina, Italy; 2 Institute for the Study of Nanostructured Materials, ISMN—CNR, Palermo, c/o Department of ChiBioFarAm, University of Messina, Viale F. Stagno d’Alcontres 31, Vill. S. Agata, 98166 Messina, Italy; 3 Noxorsokem Group Srl, Via Udine 46, SS 13, 33080 Cusano di Zoppola, Italy; 4 Department of ChiBioFarAm, University of Messina, Viale F. Stagno d’Alcontres 31, Vill. S. Agata, 98166 Messina, Italy; 5 Institute for Polymers, Composites and Biomaterials, CNR—IPCB, Via Paolo Gaifami 18, 95126 Catania, Italy

**Keywords:** sol–gel, phytic acid, (3-glycidyloxypropyl)trimethoxysilane, graphene oxide, eco-friendly coatings, nanohybrid anticorrosive coatings

## Abstract

In the past few years, corrosion protection of metal materials has become a global challenge, due to its great economic importance. For this reason, various methods have been developed to inhibit the corrosion process, such as surface treatment approaches, by employing corrosion inhibitors through the deposition of opportunely designed functional coatings, employed to preserve from corrosion damages metallic substrates. Recently, among these techniques and in order to avoid the toxic chromate-based pre-treatment coatings, silane-based coatings and films loaded with organic and inorganic corrosion inhibitors have been widely used in corrosion mitigation water-based surface treatment. In this study, the synthetic approach was devoted to create an embedded, hosted, waterborne, and eco-friendly matrix, obtained by use of the sol–gel technique, through the reaction of functional alkoxysilane cross-linking precursors, namely (3-glycidyloxypropyl)trimethoxysilane (GPTMS) and (3-aminopropyl)triethoxysilane (APTES), in the presence of graphene oxide (GO) intercalated with natural and non-toxic phytic acid (PA) molecules. As a matter of fact, all experimental results from FT-IR spectroscopy, UV–Vis analysis, and SEM confirmed that PA molecules were successfully decorated on GO. Furthermore, polarization measurements and a neutral salt spray test were used to evaluate the anticorrosive performance on aluminum and steel substrates, thus showing that the GO-PA nanofiller improved the barrier and corrosion protection properties of the developed functional silane-based coatings.

## 1. Introduction

Metal’s corrosive tendency in a harsh environment has led to huge economic losses and serious safety accidents [1]. To increase metal corrosion resistance, several efficient corrosion prevention methods have been produced, such as metal material design and treatment [2], processing with corrosion inhibitors [3], cathodic protection [4], and application of coatings [5,6,7,8,9,10,11].

Anticorrosion coatings, in particular, polymeric hybrid or nanostructured coatings, have up, until now, emerged as the most well-liked and successful methods of protecting metal from corrosion, due to their clear benefits, including their wide adaptability, economic savings, simple manufacturing, and routine maintenance. Enhancing the barrier property is of primary importance, in order to improve the anticorrosion capability of the developed surface coating. The use of nanofillers in coating production, such as mica [12] glass flakes [13], and montmorillonite [14], can lead to the enhancement of their protective performances. Graphene, a well-known two-dimensional (2D) nanomaterial, featuring one-atom thickness, with a nanosheet-like structure, is widely studied for its applications as a nanofiller in anticorrosive coating preparation. As has been well-demonstrated, it features protective performances, enhanced mechanical properties, high specific surface area, and stability [15,16,17,18,19,20,21,22,23,24,25]. However, due to π–π stacking, graphene sheets may aggregate easily, making it challenging to disperse them uniformly in a polymeric matrix to form a physical protective layer. Unfortunately, graphene nanomaterials are contentious for their broad use in anti-corrosion applications, due to both the challenging water dispersion approaches and strong electrical conductivity [26]. 

Due to its excellent properties and scalable manufacturing capacity [27], another form of graphene-based nanostructure, graphene oxide (GO), has caught the interest of researchers as a potential solution to these disadvantages [28,29]. In particular, GO is a layered nonconductive hydrophilic carbon nanomaterial that could be easily obtained by the oxidation and exfoliation of graphite by oxidant agents (Hummer’s technique KMnO_4_, NaNO_3_, and H_2_SO_4_) [30]. Due to its negatively-charged surface, GO features a high capacity for the adsorption and intercalation of various molecules via physical and/or chemical forces, such as electrostatic and hydrophobic interactions. The use of GO as a host material, as well as its incorporation in host materials, are the two major approaches for functionalizing GO and generating nanocomposites or nanohybrids. In the first scenario, multiple functionalization techniques are used to decorate GO surface [31]. Moreover, graphene oxide not only avoids the two aforementioned limits of graphene, but also preserves the key properties of this nanomaterial, thus leading it to finding applications in a wide range of sectors, such as biomedical [32], sensors [33], analytical techniques [34], and environmental remediation [35]. As a result, it might serve as a suitable alternative to graphene-based products in anticorrosion treatments. The increased surface area and Van der Waals interactions, however, produce a substantial aggregation of GO nanosheets in polymer-based coating applications, as well, which results in not enough barrier performances of the final GO/polymer coating system [36,37,38]. Thus, the functionalization of GO is necessary for enhancing its dispersion in polymer-based coatings. In addition, GO has several organic functional groups that characterize its surface and edges (such as the –OH, –COOH, and epoxy groups) and serve as active sites for chemical modifications [39,40,41,42,43,44,45]. A particularly successful approach for solving this issue is the covalent and/or noncovalent chemical modification of GO.

Since the functional groups of GO represent suitable active sites for interacting with other composites, their reactivity with the polymer matrix is increased. Although incorporating surface-functionalized nano-fillers considerably improves the coatings’ ability to resist against corrosion, they hardly show any effective “self-healing” characteristics for nanocomposites coatings when corrosion degradation first appears. In this regard, phytic acid (PA), an eco-friendly plant seed and cereal grains derivative, characterized by an organic coordination structure, features different notable properties, such as very good water solubility, non-toxicity, and biocompatibility, with applications in different sectors from food to cosmetic preparation and water treatment [46,47,48,49]. 

The molecular structure of PA, C_2_H_12_O_24_P_6_, exhibits 6 phosphate groups and 24 oxygen atoms, where every carbon atom of the cyclo-hexamehexol ring has a linked phosphate group [50,51]. With its six phosphoric acid groups, PA might chelate with metal ions, such as the Fe^2+^, Ca^2+^, Mg^2+^, and Zn^2+^ ions [52,53,54], and lead to the formation of a chemical conversion film on the metal surface that can significantly reduce the direct contact between corrosive media and metal substrates. Due to this peculiar behavior, the PA may offer efficient and environmentally friendly anti-corrosion capabilities for preventing metal corrosion [55,56,57,58]. Liu et al. [59] demonstrated that the PA conversion surface might give magnesium alloys effective anti-corrosion performance. Meanwhile, Gao et al. [60] studied creating cerium and PA-containing composite films. Due to the synergistic action of the cerium and PA on the metal’s surface, they demonstrated the highest anti-corrosion efficacy among all coatings. In the pretreatment process of metal corrosion protection, PA is, therefore, frequently utilized as a sustainable corrosion inhibitor [61,62]. Finally, functionalizing GO with PA can be a promising approach to enhancing the anticorrosion performance of the coatings. 

In this work, PA was employed to covalently modify GO, with the aim of preparing a graphene oxide-pythic acid functional nanofiller (GO-PA) [63,64,65,66,67]. The decoration of the GO surface with PA leads the functional GO-PA nanofiller to an enhanced water dispersion stability, with the corrosion inhibition capability of pristine GO. Subsequently, GO-PA was used as nanofiller for preparing silane- and water-based anticorrosion multicomponent coating, featuring the synergistic anticorrosion performances of both GO and PA, in combination with the well-known inertness of silane cross-linkers. As a matter of fact, in sol–gel chemistry, it is widely employed for the preparation of anticorrosion protective coatings, thanks to its versatility and easy execution, thus leading to surfaces with enhanced mechanical strength, chemical stability, and thermal resistance [68]. In this regard, different nanofillers and approaches were recently employed to enhance and induce anticorrosion performances on sol–gel-based protective coatings, and some of them are listed in Table 1.

Moreover, the sheet-like structure of GO seems to play the role of a physical barrier, exploiting its anticorrosion performances. On the other hand, GO decorated with PA, thanks to the chelation properties of metal surfaces and, in particular, iron can act with a synergistic effect as an effective functional protective anti-corrosion layer. In this study, the anti-corrosion properties of the silane–based coating loaded with GO-PA were studied via the Tafel polarization and neutral salt spray (NSS) tests. All experimental results showed that the anti-corrosion performance of the obtained waterborne eco-sustainable sol–gel coating, based on phytic acid intercalated graphene oxide, was significantly enhanced, thus placing itself on a path towards a valuable, eco-friendly, and simple approach for obtaining efficient anti-corrosive and protective coatings for different metal-based application fields.

## 2. Results and Discussions

### 2.1. Synthesis of the Waterborne Multicomponent Coatings

**Figure 1:**
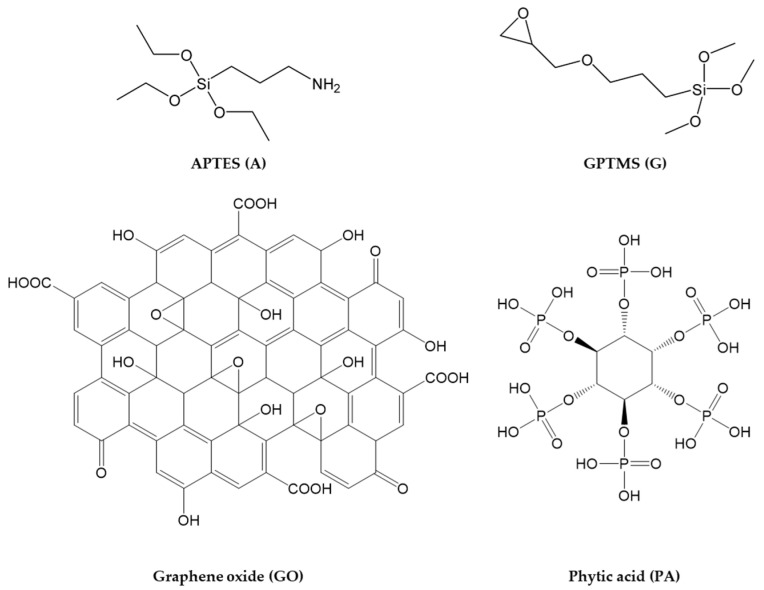
Functional molecules employed for the preparation of the waterborne anticorrosive coatings.

The four functional molecules shown in Figure 1 were opportunely selected and employed as synthons for the design and development of a multicomponent anticorrosive waterborne sol–gel coating featuring their synergistic effect.

In particular, the anticorrosive performances of graphene oxide intercalated phytic acid are well-known in the literature [79,80,81,82]. Moreover, the sol–gel process is widely employed for the preparation of thin films, thanks to their chemical inertness, outstanding resistance, and mechanical properties [83,84,85,86].

**Figure 2:**
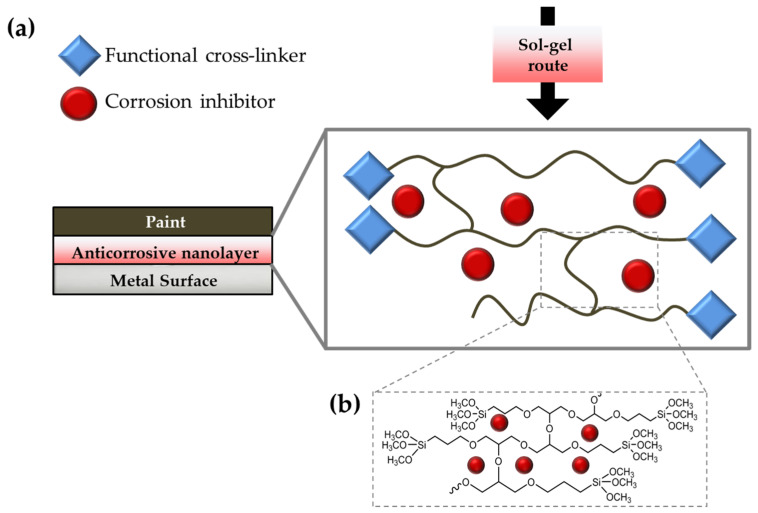
Cross-linking behavior schematization of the functional alkoxysilanes with the presence of the anticorrosive nanofillers (**a**) and detail of the GPTMS cross-linking reaction (**b**).

In this work, two alkoxysilanes (3-aminopropyl)triethoxysilane (hereafter APTES or A) and (3-Glycidyloxypropyl)trimethoxysilane (hereafter GPTMS or G)) were carefully chosen as cross-linkers, in order to achieve the preparation of two types of functional coatings [87,88]. In detail, GPTMS has been widely employed for coating applications, thanks to its two different functional ends [89,90,91,92], i.e., a binding trimethoxysilyl group and anchoring epoxy ring; the latter may undergo an epoxy-ring opening reaction, due to the nucleophilic substitution reaction of the carboxylic groups, either in the GO-PA nanofiller, while the silane end will lead through the usual silane stages of hydrolysis and, subsequently, condensation to the expected alkoxysilane polymerization towards the final development of a polyethylene oxide network (PEO) (see Figure 2b) [93,94]. Meanwhile, nucleophilic substitution reactions among the primary amino-silane APTES lead to the preparation of a reactive sol that can covalently bond the functional carboxylic nanofillers. The obtained coating may be used as a primer layer between the metal surface and an external top paint (Figure 2a).

The hydrolysis and condensations steps, characterizing the sol–gel processes, could promote their anchoring to specific substrates. In particular, covalent connections can be formed between the–OH groups of the metallic substrates (obtained by alkaline or acid surface-pretreatment) and hydrolyzed –OCH_2_CH_3_ or –OCH_3_ groups of the two functional silanes. Additionally, when metal-siloxane linkages are formed in a temperature-driven hydrolysis/condensation event, an enhanced adhesion may be observed. The formation of additional silanol groups (Si–OH) into –Si–O–Si– siloxane chains could also establish a strong network layer that serves as a powerful barrier towards aggressive species [95], as well as as potential sites for the adhesion of a subsequent layer of top paint.

### 2.2. GO and GO-PA Nanofillers

#### 2.2.1. Dispersibility of GO-PA

**Figure 3:**
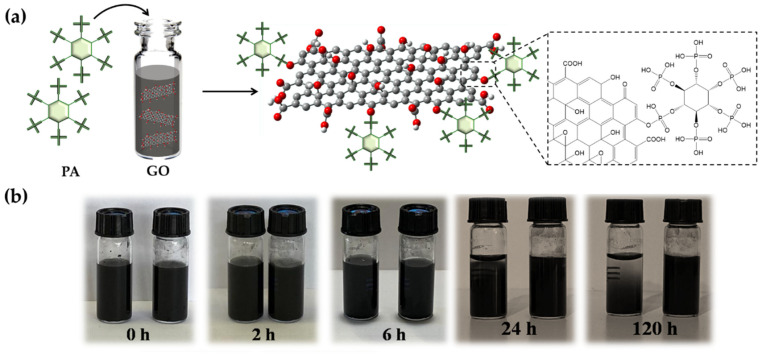
Probable reactions during PA intercalation of GO (**a**). Dispersion of GO (**left**) and GO-PA (**right**) (**b**) in water after various storage periods.

The potential responses to the PA intercalation of GO are depicted in Figure 3a. The epoxy group on the GO surface and PA establish a covalent link, leading to the formation of the functional nanofiller. The sedimentation test in Figure 3 illustrates the GO-PA and GO ability to disperse in aqueous solutions. In this investigation, GO and GO-PA were ultrasonically dispersed in water for half an hour, and the stable dispersion was subsequently kept in storage for several days without disturbance. It can be easily observed that GO-PA is better dispersed than GO in water. The GO-PA solution did not clearly stratify after 5 days, as seen in Figure 3b, demonstrating that the PA-modified GO has improved the water dispersion capacities.

The enhanced water dispersion capacity of GO-PA should be attributed to the successful modification of the GO surface with PA. Therefore, this evidence also provides optimistic conditions for GO-PA applications in the development of coatings featuring anticorrosive performances. 

**Figure 4:**
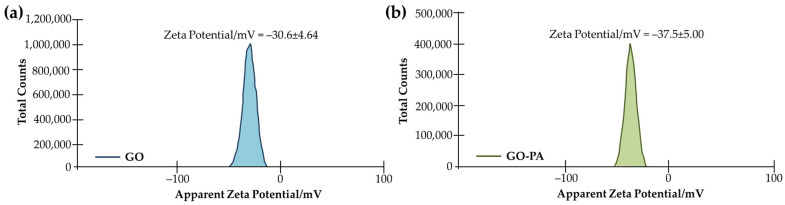
Average zeta potentials of GO (**a**) and GO-PA (**b**) in water dispersion.

The analysis of the zeta potentials of GO and GO-PA in water dispersion was performed with the zeta potential test (both are 5 mg mL^−1^). To ensure that the results were accurate, two tests for each sample were performed. The findings (see Figure 4) indicate a −30.6 mV average zeta potential for GO. Meanwhile, for GO-PA, a notable negatively shifted average zeta potential, from −30.6 to −37.5 mV, was revealed. This result, together with the dispersion test, suggests better GO dispersion stability, due to PA modification, which is explained by the grafting of negatively-charged phosphate groups from PA molecules onto the GO surface [81].

#### 2.2.2. FT-IR Analysis

**Figure 5:**
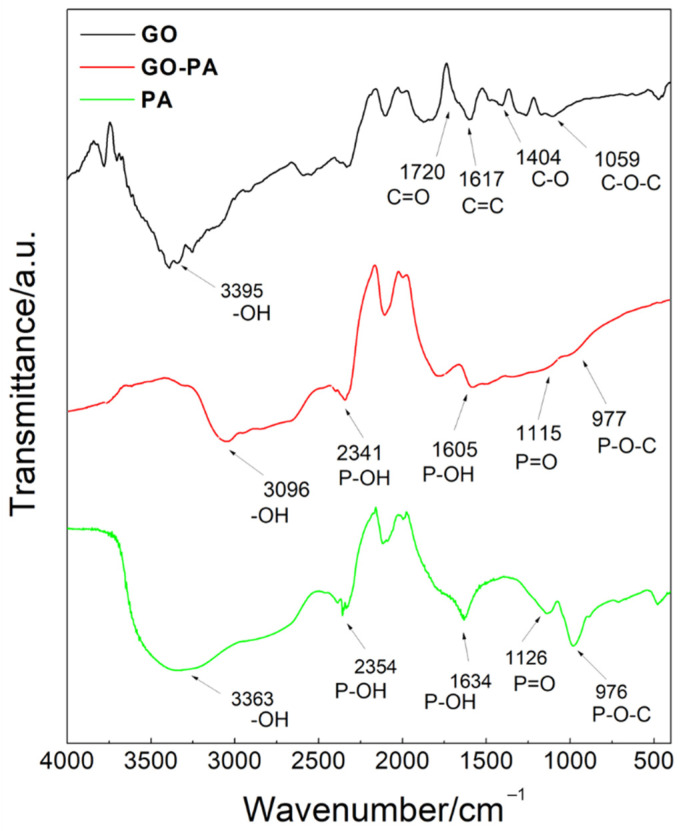
ATR-FT-IR spectra of PA, GO, and GO-PA.

The chemical structure of PA, GO, and GO-PA have been studied by FT-IR (Figure 5) spectroscopy, in order to demonstrate the successful preparation of the GO-PA nanofiller. In the spectrum of PA (green line), its characteristic peaks appear at 976, 1126, 2354–1634, and 3363 cm^−1^, and they were attributed to the P-O-C, P=O, P-OH, and –OH stretching vibrations, respectively [96,97].

The pristine GO nanosheets (black line) showed peaks located at 1059, 1404, and 3395 cm^−1^, which can be assigned to the stretching vibration of the epoxide C-O-C, hydroxyl C-O, and -OH groups, respectively [98,99,100].

Meanwhile, the absorption peaks appearing at 1720 and 1617 cm^−1^ correspond to the C=O stretching vibration and C=C from the benzene ring skeleton structure, indicating the reserved unoxidized graphitic domains [101].

The red line in Figure 5 represents the FT-IR GO-PA spectrum, where the characteristic absorption signals of both GO and PA can be observed. In particular, the peaks located at 977, 1115, and 1605–2341 cm^−1^ are assigned to the P-O-C, P=O, and P-OH groups of PA. At 1720 cm^−1^, it is also possible to notice a stronger C=O stretching vibration band, compared to GO spectra, provided that the GO was successfully functionalized with PA. Moreover, the shift of –OH peak at 3096 cm^−1^ can be attributed to the formation of the association links between many PA hydroxyl groups [102,103]. It is also important to notice that, in the GO-PA spectrum, the addition of several hydroxyl groups from the PA molecules to the GO surface is responsible for the shifted signal of the OH signal. Additionally, it can be attributed to the increased electrostatic interactions and hydrogen bonding between hydroxyl units of the GO and PA molecules [104]. Therefore, these results confirm the synthesis of the GO-PA functional nanofiller.

#### 2.2.3. UV–Vis Spectral Changes

**Figure 6:**
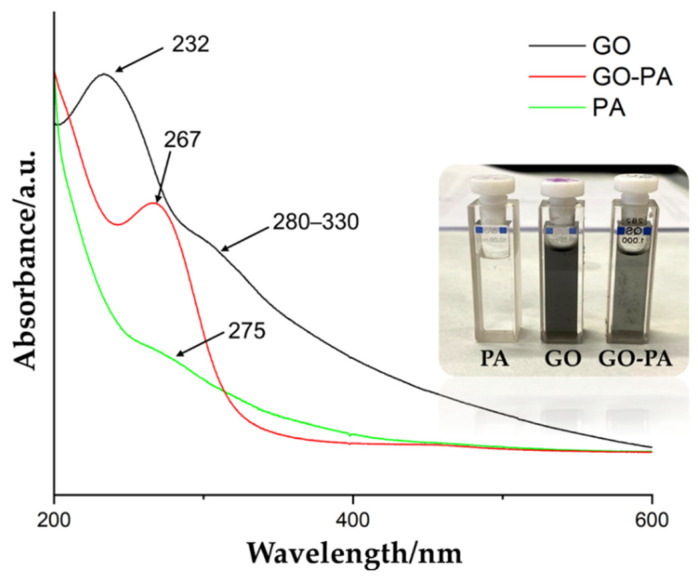
UV–Vis spectra of PA, GO, and GO-PA water dispersions and their visual appearance in the inset.

Figure 6 shows the comparison of the UV–Vis spectra of PA, GO, and GO-PA, in order to support the successful obtaining of the functional GO-PA nanofiller. The green line represents the PA spectra, showing its characteristic peak at 275 nm. The GO spectra (black line) is characterized by a relevant peak at 232 nm and shoulder-like peak at 280–330 nm, indicating the aromatic C=C π → π * and C=O n → π * transitions, respectively [105,106].

After the functionalization of GO with PA, a new characteristic peak was visible at 267 nm (red line), which is attributed to the absorption of the PA molecule and intercalation of the GO nanosheets. Therefore, the successful functionalization of GO with PA, is again confirmed. Additionally, Figure 6 shows the visual appearance of the water suspensions of GO, PA, and GO-PA (inset). It is possible to observe a change in their color from the brown of GO to the black of GO-PA, thus proving the effective grafting of PA on the GO surface [105].

#### 2.2.4. Raman Spectroscopy

**Figure 7:**
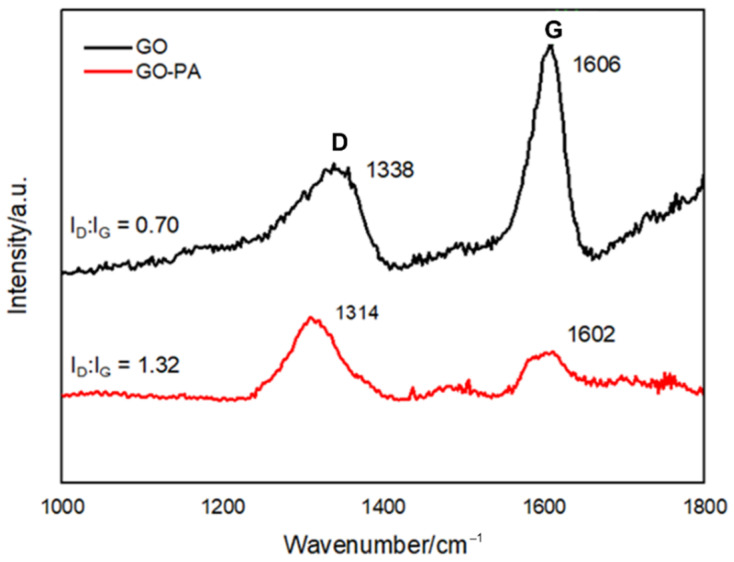
Raman spectra of GO and GO-PA nanofillers reporting the D and G peaks intensity ratio, respectively.

Raman spectroscopy was used to characterize the surface defect degree and degree of alteration of GO-PA, as well as to explore structural variation of carbon-based nanomaterials. Figure 7 displays the Raman spectra of GO and GO-PA, focusing on the D and G peaks (typical characteristic reference peaks) that reflect the structural changes of graphene. Since defects and distortions of the sp^2^ domains are common to all sp^2^ carbon lattices and result from the stretching of the C–C bond, the broadened D peak, which is related, indicates the size reduction of the in-plane sp^2^ domains of the graphite and prove the successful obtaining of GO [107].

In the GO spectrum, at 1338 and 1606 cm^−1^, it is possible to observe the D and G band characteristics of GO (black line), referred to the sp^3^ carbon atom vibration deriving from the functional groups and sp^2^ carbon atoms of the in-plane vibration, respectively. A shift of the G peak from 1606 (GO) to 1602 cm^−1^ (GO-PA), is visible, indicating the effective functionalization of GO with PA [99].

The I_D_/I_G_ ratio for GO, which measures the degree of disorder and is inversely correlated with the average size of the sp^2^ clusters, was also determined. In this regard, the calculation results show that, compared with GO, the intensity ratio between the D and G peaks of GO-PA reveals a slight improvement from 0.70 (GO) to 1.32 (GO-PA), due to the increase of the surface defect density of GO, for the PA grafting [108,109,110,111].

#### 2.2.5. X-ray Diffraction Spectroscopy (XRD)

**Figure 8:**
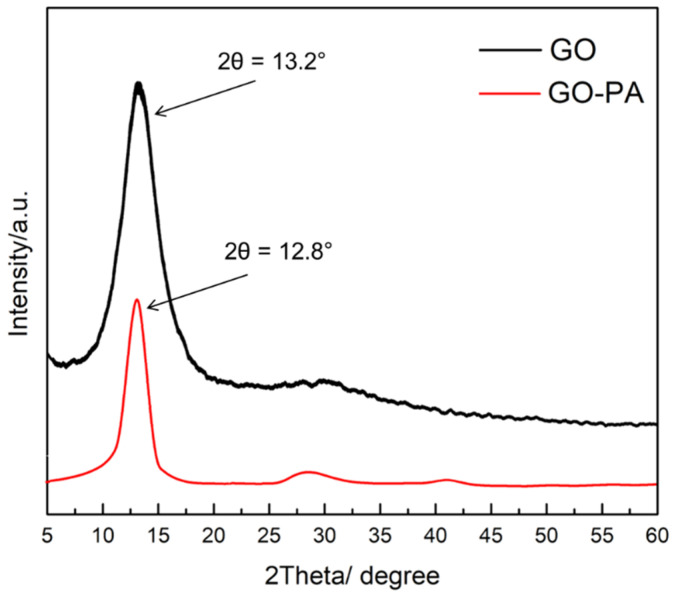
XRD patterns for GO and PA-GO nanomaterials.

The XRD spectra of GO and GO-PA are shown in Figure 8. The GO XRD pattern showed a strong diffraction peak of the (001) plane of GO at 2θ = 13.2°, revealing that graphene oxide basal planes were oriented most favorably parallel to the sample plane, therefore demonstrating that the crystal structure of GO was complete and ordered [112]. An interlayer distance of 6.7 Å was also observed, corresponding to the interlamellar spacing of the GO. This value of interlamellar spacing is attributable to GO in its dry state, as the completely hydrated GO’s layer distance can vary by up to 12° [113]. Subsequently to the modification, the diffraction peak of GO-PA is approximately at 12.8°, with an interlayer of 6.9 Å, which can be attributed to the intercalation of the PA molecules through the GO nanosheets, thus indicating that PA molecules diffusion into the GO nanosheets leads to the partial exfoliation of the nanosheets. It is, in fact, reported that PA grafted on the GO surface may reduce the π–π stacking interactions between the sp^2^ structure of GO nanosheets [82].

According to these findings, with the higher interlamellar spacing between the GO layers, it is possible to assess whether PA was successfully incorporated into GO nanosheets.

#### 2.2.6. Scanning Electron Microscopy (SEM)

**Figure 9:**
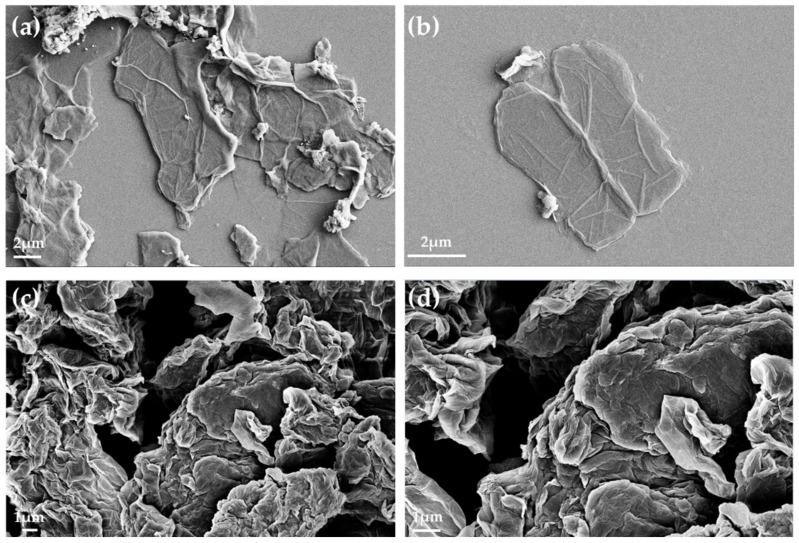
SEM images for GO (**a**,**b**) and GO-PA (**c**,**d**).

The morphology of the GO and GO-PA was characterized by scanning electron microscopy (SEM), in order to gain insight regarding the effects of the PA intercalation on GO. SEM images for the GO and GO-PA were displayed in Figure 9a–d. 

Figure 9a, and the relative magnification in Figure 9b, with micrographs of the GO powder, show the characteristic sheet structure of the few-layer graphene oxide nanomaterial, distinguished by its smooth surface and wrinkled texture [114]. Additionally, the borders of the sheets, including the kinked and wrinkled portions, can be seen on such few-layer GO films, which are occasionally folded or continuous. However, Figure 9c, and the relative magnification in Figure 9d of the modified GO powder (GO-PA), demonstrate that the GO intercalated PA nanofiller is characterized by a more granular morphology than pure GO. Moreover, in the GO-PA matrix, the micrometric agglomerates of the rough folds are visible in the structure of the single sheet. 

These characteristics are further proof of the successful decoration of GO surface with PA molecules [115].

### 2.3. GO-PA Sol–Gel Nanohybrid Coatings

#### 2.3.1. Optical Microscopy and Roughness Measurement

**Figure 10:**
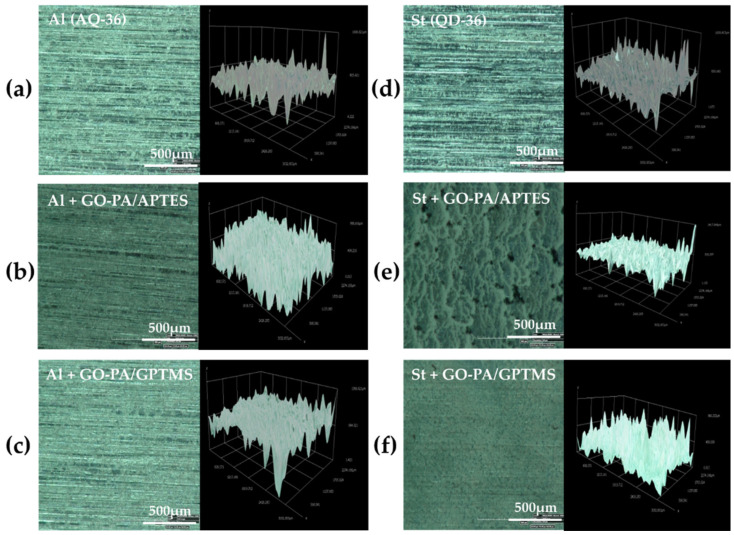
Optical microscopy images of GO-PA/APTES (**b**,**e**) and GO-PA/GPTMS (**c**,**f**) coatings deposited on the aluminum (**a**) and steel (**d**) standard panels, with 3D images of the roughness of the analyzed samples.

In order to investigate the morphology of the obtained sol–gel nanohybrid coatings with GO-PA nanofillers, the optical microscopy was employed, to observe in particular the variations in terms of roughness (see Figure 10a–f).

**Figure 11:**
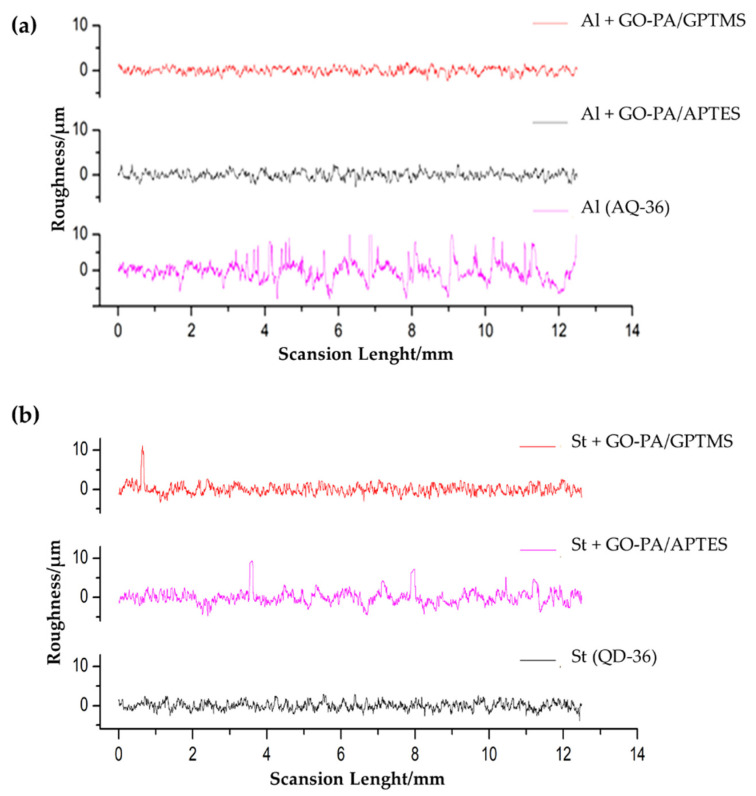
Surface roughness profiles of the different coatings deposited on aluminum (**a**) and steel (**b**).

The images show that, in the case of aluminum (see Figure 11a,b), GO-PA coating treatment has no apparent effect on the surface morphology of the substrates, and the roughness increases only for the GO-PA/APTES coating, as proven by the roughness profile values collected in Table 2. This increased roughness may enhance the subsequent attachment of the paint to the substrate, as demonstrated by adhesion tests, thus providing promising benefits, in terms of protective activity. For the GO-PA/GPTMS coating, the average roughness remains almost constant. On the other hand, in the case of steel (see Figure 11d–f), the GO-PA coating treatment seems to alter the surface morphology of the substrates, with the formation of circular pores for the GO-PA/APTES coating absent in the starting sample. Indeed, from the data shown in Table 2, the steel substrates treated with the GO-PA coatings show a slight decrease in roughness, which is higher in the sample GO-PA/APTES, with an Ra of 5.09 µm and Rz of 12.55 µm; it is lower in the sample GO-PA/GPTMS, with an Ra of 5.48 µm and Rz of 12.73 µm.

#### 2.3.2. SEM-EDX

**Figure 12:**
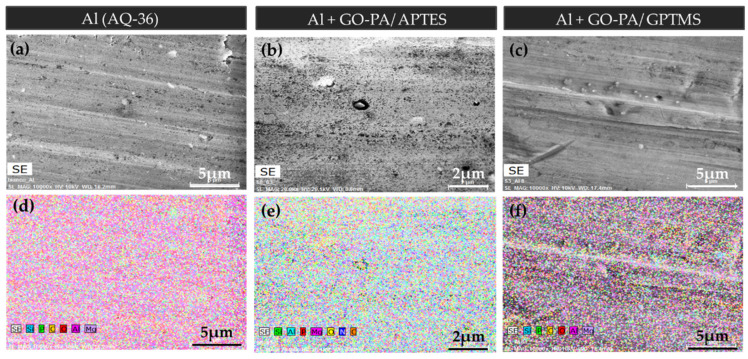
SEM images of no-treated aluminum substrate (**a**) and aluminum substrates treated with GO-PA/APTES (**b**) and GO-PA/GPTMS (**c**) coatings. EDX mapping of C, O, and P elements of the analyzed samples (**d**–**f**).

EDX mapping based on SEM (see Figure 12a–c) was used to additionally investigate the dispersion of the GO-PA nanofiller in GPTMS- and APTES-based matrices on aluminum and steel substrates. According to the EDX mappings, which are displayed in Figure 12 d–f, the C, O, and P elements are evenly distributed across the surface of GO-PA. The fact that the P element is evenly distributed across the entire surface demonstrates that the PA is distributed uniformly. In conclusion, the GO-PA nanofiller exhibits well-dispersed performance in both silane-based coating matrices, indicating that it can greatly improve the coatings’ barrier properties. This effect will be further examined in the characterization of the anti-corrosion performances.

#### 2.3.3. Adhesion Measurements: Pull-Off and Cross-Cut Test

Evidence of excellent adhesion of paint after the treatment of aluminum and steel substrates with the GO-PA/GPTMS and GO-PA/APTES coatings was attained through coatings’ thickness and adhesion strength evaluations.

**Figure 13:**
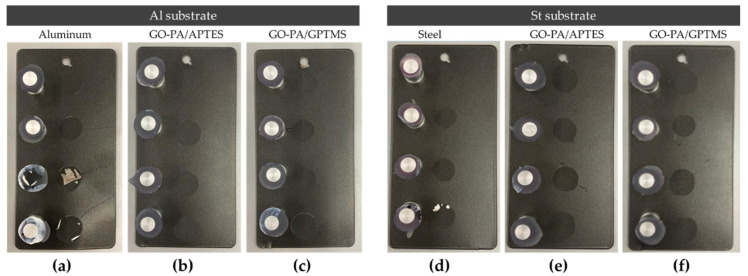
Pull-off adhesion test of paint on aluminum (**a**–**c**) and steel (**d**–**f**) substrates treated with GO-PA/GPTMS (**c**,**f**) and GO-PA/APTES (**b**,**e**) coatings.

Pull-off adhesion and cross-cut tests were performed to investigate the effect of GO-PA-based coatings (GO-PA/APTES and GO-PA/GPTMS) on the adhesion strength of a commercial paint on the surface of aluminum and steel substrates. Figure 13 reveals that treatment with GO-PA coatings can effectively improve the adhesion strength between the paint and aluminum or steel substrates. The phosphate groups of PA have very good metal ion chelation capacity, according to the literature [116]. Therefore, GO-PA-based coatings are able to interact directly with the substrate.

**Figure 14:**
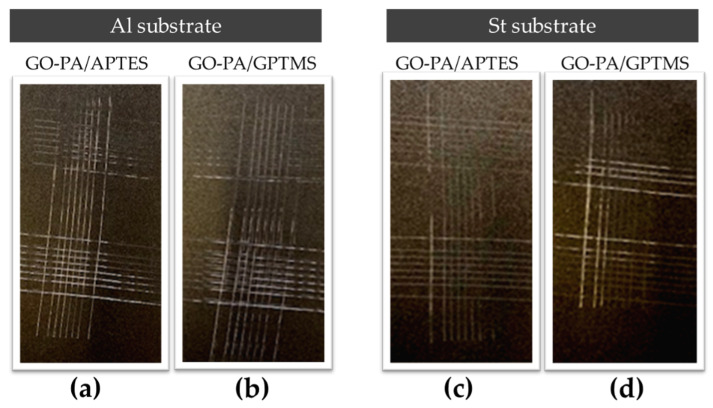
Adhesion cross-cut test of paint on aluminum (**a**,**b**) and steel (**c**,**d**) substrates treated with GO-PA/GPTMS (**b**,**d**) and GO-PA/APTES (**a**,**c**) coatings.

The maximum adhesion values for both substrates, ISO 0 and ASTM 5B, were obtained via the cross-cut adhesion test, which provides a visual comparison technique for measuring paint adhesion integrity, as verified against ISO 2409 and ASTM D 3359 standards. Additionally, an ASTM 5B value is provided to a coating if all of the edges of the cut are entirely smooth and none of the lattice squares that were generated as a result of the cuts are detached [117]. According to Valli, who was discussing thin, durable coatings for steel protection, adherence is a coating’s most crucial quality for future effective use [118]. According to the reference standards, none of the squares in the lattice are detached, and the edges of the cuts in Figure 14a–d are entirely flat.

This coatings’ remarkable qualities, which cannot interfere with the adhesion of the following paint treatment, are demonstrated by their exceptionally excellent high scratch resistance. As a result, a notable improvement is made to the coatings’ overall adhesion capacity. 

**Figure 15:**
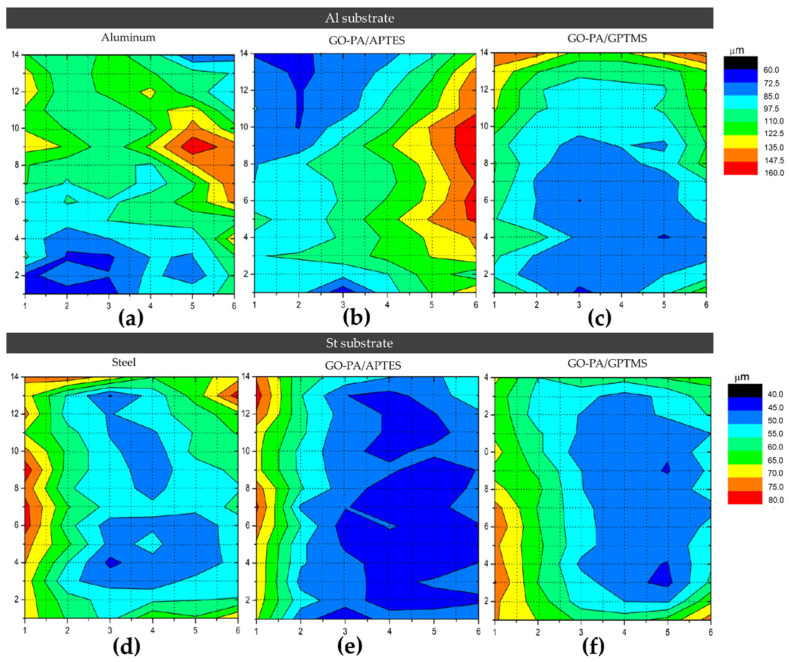
A 14 × 6 grid chromatic map of the metallic specimens in AQ-36 aluminum (**a**–**c**) and QD-36 steel (**d**–**f**) coated with paint.

For AQ-36 aluminum substrates, the chromatic map in Figure 15a–c indicates average thicknesses of about 102 and 160 μm for the GO-PA/APTES and GO-PA/GPTMS coatings, respectively. As for the QD-36 steel substrates, the chromatic map in Figure 15d–f indicates average thicknesses of about 62.5 and 80 μm for the GO-PA/APTES and GO-PA/GPTMS coatings, respectively. The exact values of the thickness of the coating samples are shown in Table 3. 

#### 2.3.4. Evaluation of Anticorrosive Performance

Polarization measurements and salt spray test for painted steel and aluminum specimens were performed. 

**Figure 16:**
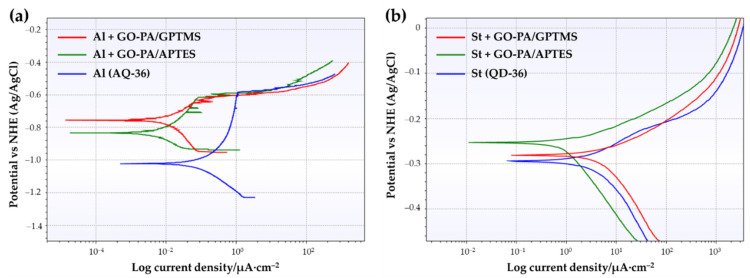
Curves of Tafel polarization, related to different coating samples (GO-PA/GPTMS and GO-PA/APTES) on aluminum (**a**) and steel substrates (**b**).

One approach for evaluating coating anticorrosion performance is the polarization test, which measures the coating’s corrosive potential (E_corr_), corrosion current density (i_corr_), polarization resistance (R_p_), and corrosion rate (CR). Generally, the shift through a more positive value for E_corr_ and lowering of i_corr,_ compared to the untreated sample, indicates improved anticorrosion capabilities [119]. The corrosion protective efficiency (PE), based on the corrosion current density of bare AQ-36 aluminum and QD-36 steel, quantifies the corrosion protective ability of the different coatings, according to the following equation [120,121]:

(1) $$\mathrm{PE}(\%)\;=\;\frac{i_{\mathrm{corr}}-i_{\mathrm{corr}}^{0}}{i_{\mathrm{corr}}^{0}}\times \;100\%$$

where i_corr_ represents the corrosion current density of the coating, and $i_{\mathrm{corr}}^{0}$ represents the corrosion current density of bare AQ-36 aluminum and DQ-36 steel. Tafel polarization curves for the GO-PA-based coating samples were obtained in 3.5 wt.% NaCl aqueous solution, and they are shown in Figure 16a,b. Table 4 and Table 5 provide a summary of the related Tafel curve’s calculation parameters.

As shown in Table 5, GO-PA/GPTMS and GO-PA/APTES coatings applied on steel substrates exhibit more positive E_corr_ and lower i_corr_, with respect to that of bare QD-36, with weak corrosion protective efficiencies (PE%) of 41.06 and 46.03%. The values of E_corr_ for St (AQ-36), St + GO-PA/GPTMS, and St + GO-PA/APTES are −0.309 V, −0.281 V, and −0.253 V, although the values of i_corr_ are 3.02 × 10^−5^ A/cm^2^, 1.63 × 10^−5^ A/cm^2^, and 1.68 × 10^−5^ A/cm^2^, respectively. In contrast, as shown in Table 4, for aluminum substrates, the GO-PA/GPTMS and GO-PA/APTES coatings exhibited more positive E_corr_ and lower i_corr_, with respect to that of bare AQ-36, with a good corrosion protective efficiency (PE%) of 95.62 and 98.97 %. The values of E_corr_ for Al (AQ-36), GO-PA/GPTMS, and GO-PA/APTES were −1.022, −0.756, and −0.834 V, while the values of i_corr_ were 4.29 × 10^−7^, 1.88 × 10^−8^, and 4.44 × 10^−9^ A/cm^2^, respectively. In both cases, the GO-PA/APTES coating showed the lowest i_corr_ values, as well as more positive E_corr_ than the other coating. Lower corrosion density and a more positive corrosion potential suggest that the GO-PA nanomaterial, used as filler in silane-based matrix, positively affects the corrosion resistance of the final coatings, thus indicating a final certain corrosion-inhibition property.

**Figure 17:**
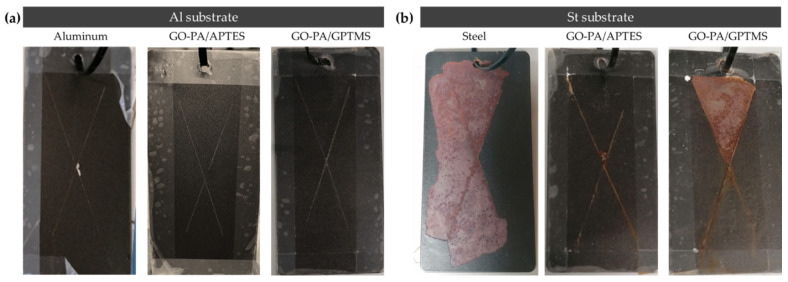
Visual images of painted aluminum (**a**) and steel (**b**) substrates without GO-PA nanofiller and with GO-PA nanofiller (as GO-PA/GPTMS and GO-PA/APTES coatings) after salt spray test (aluminum: 900 h; coated Al 1300 h; steel: 50 h; coated St: 400 h).

The salt spray test was performed to further analyze the corrosion protection capability of the GO-PA-based coating samples. Figure 17 shows visual pictures of the painted aluminum (a) and steel (b) panels treated with PA-GO based coating (GO-PA/GPTMS and GO-PA/APTES) and untreated samples after exposure in neutral salt spray environment. 

The figure shows that different substrates exhibited varying extents of corrosion areas, following the application of equivalent GO-PA coatings, in agreement with the polarization measurement indicated above. For the steel QD-36 substrates, the corrosion product accumulation and paint peeling were visible around the scratch zone, thus showing the poor anti-corrosion ability of the GO-PA-based coatings. Among them, the corrosion of the St + GO-PA/GPTMS was the most serious, while the corrosion spots of St + GO-PA/APTES was relatively minimal. This could be due to the incompatibility of the coating with the steel substrate or with the paint, probably due to the acidic nature of the nanofiller, thus reducing the anti-corrosion performance of the coatings. This behavior could be counteracted in the future by buffering the coating solution, thus increasing the pH value and improving corrosion inhibition. In contrast, the GO-PA coatings exhibit improved corrosion resistance for aluminum substrate, which does not lead to any accumulation products and coating delamination among all coating samples, increasing the specimen’s resistance to NSS from 900 h of the bare aluminum to 1300 h of the coated ones. 

## 3. Materials and Methods

### 3.1. Materials

Sodium nitrite, hydrochloric acid (37%), hydrogen peroxide solution 35% (*w*/*w*) in H_2_O, potassium permanganate, sulfuric acid (98%), phytic acid 50% (*w*/*w*) in H_2_O (PA), (3-aminopropyl)triethoxysilane (A, or APTES), (3-glycidyloxypropyl)trimethoxysilane (G, or GPTMS), and graphite powder with the grain size < 20 μm were obtained from Merck (Darmstadt, Germany). They were all purchased at the highest purity level and used as received, without any further purification. Deionized water was prepared using corresponding equipment. 

### 3.2. Synthesis of Graphene Oxide

The GO nanosheets were made using a modified version of Hummer’s method, as follows: (i) 5 g of graphite powder was added in 120 mL of concentrated H_2_SO_4_ and stirred for 2 h; (ii) 15 g of KMnO_4_ and 2.5 g of NaNO_3_ were added slowly into the mixture and stirred for 72 h; (iii) the obtained mixture was diluted with 600 mL of deionized water and H_2_O_2_, added dropwise after 30 min, obtaining a yellow-brown solution; (iv) subsequently, the raw product was firstly washed with a 1:10 HCl solution and deionized water, and then it was centrifugated for 3 min at 3000 rpm, until reaching neutral pH, in order to obtain the final GO. 

### 3.3. Functionalization of GO with PA

Firstly, GO nanosheets (0.3 g) were poured into deionized water (200 mL) and then placed in an ice bath sonicator for 30 min to obtain a uniform dispersion of GO. Subsequently, a mixture of GO and PA (6 g) was prepared and sonicated for 2 h under stirring and at room temperature. Therefore, after the conclusion of the reaction, PA-modified GO (GO-PA) was obtained, and the excessive non-reacted PA was removed by several washings using deionized water. Finally, when the neutral pH was reached, the cleaned PA-modified GO was uniformly dispersed in deionized water by sonication, dried in oven at 70 °C for 12 h, and cell pulverized for storage. 

### 3.4. Preparation of Nanohybrid Coatings/Hybrid Sol

The nanohybrid coatings were obtained through the following steps. Aqueous suspension of GO-PA was mixed with an alkoxysilane water-based solution, prepared by using GPTMS or APTES as precursors. The resultant solution was vigorously stirred at 25 °C for 24 h to obtain homogeneous dispersion. This sol was used to coat aluminum and steel substrates specimens. 

### 3.5. Sample Preparation and Coating Method

In this study, Q-PANEL standard test substrates (AQ-36 aluminum and QD-36 carbon steel), whose dimensions are shown in the Table 6, were purchased from Q-Lab Corporation. In order to prepare the samples for dip-coating, they were cleaned by ultrasonic degreasing with 10% detergent solutions, DEGRIX L 420^®^ for the steel and DEGRIX L 321^®^ for the aluminum provided by NoxorSokemGroup^®^, and rinsed by demineralized water. This cleaning process thoroughly cleans the panels and removes any contaminants that might be on the surface due to preservation before use of the specimens. 

**Figure 18:**
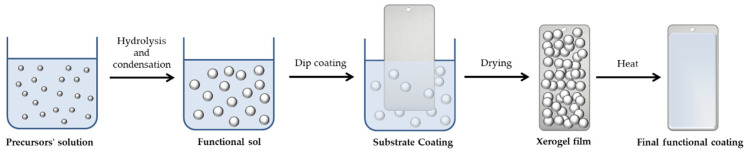
Schematization of the sol–gel process steps involving the preparation of the functional coating on metallic substrates.

The preparation of the sol–gel films involved the dip-coating of the prepared specimens in the sol solution for five minutes, then rinsing them with deionized water. Following coating application, the specimens were dried for 1 h in an oven at 60 °C before being cured for 1 h in an oven at 130 °C. A powder paint was applied to the prepared substrates and then cured at 180 °C in an industrial line. The nanohybrid coating behaves similarly to a conventional sol–gel polymeric matrix: the alkoxysilane hydrolysis and condensation reactions firstly bring the synthesis of a functional sol, which, by a dip-coating process, is employed to coat different metallic substrates; this will also lead to the formation of a xerogel film that, after a curing phase, will finally allow us to obtain the desired functional coated surface, as shown in Figure 18. 

**Figure 19:**
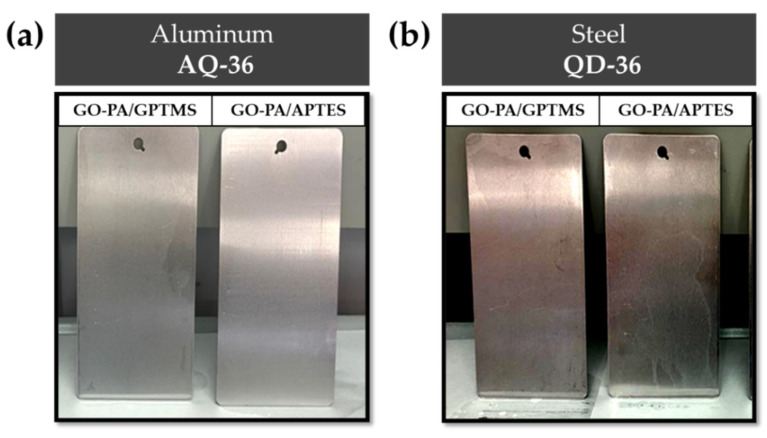
Aluminum AQ-36 panels (**a**) and QD-36 steel panels (**b**) treated with GO-PA-based coatings.

The final coated samples were stored until the tests (Figure 19). 

### 3.6. Characterizations

Fourier transform infrared (ATR-FT-IR) spectroscopy, ultraviolet–visible (UV–vis) spectroscopy, Raman spectroscopy, and X-ray diffraction (XRD) were employed to characterize the structural modification of GO after PA functionalization. 

For FT-IR analysis, a V-6600 Jasco spectrometer, including the intuitive Spectra Manager™ Suite with integrated search software solution, KnowItAll® Informatics and database JASCO Edition (JASCO Europe s.r.l., Cremella, LC, Italy), endowed with an attenuated total reflection (ATR) accessory, was employed, and the spectra of liquid samples recorded, with spectra range of 4000–500 cm^−1^ at room temperature. 

UV–vis spectroscopy (UV–Vis V-770, Jasco equipped with Standard Measurement and Analysis Programs, and Spectra Manager™ Suite Spectroscopy Software, JASCO Europe s.r.l., Cremella, Italy) was performed at 25 °C via a wide range from 200 to 800 nm. 

Raman measurement was performed to gain the Raman spectra, employing a BRAVO (Bruker Optics, Billerica, MA, USA) spectrometer, operating in the 450–3200 cm^–1^ range. The source was constituted by two lasers operating at the wavelength of 785 and 1064 nm. The scanning spectral range was 1000–3200 cm^−1^. The spot size was 10–15 micron at 10× lens.

XRD measurements were carried out by using a D8 Advance Bruker instrument (Bruker, Billerica, MA, USA) equipped with a monochromatic CuKα radiation source (40 kV, 40 mA). Bragg-Brentano theta-2theta configuration and a scanning speed of 0.1°/s were used to examine the samples in a wide range, from 10° to 80°. 

SEM micrographs were collected by a Quanta 450 FEI, with a large-field detector (LFD), on Cr-coated samples, with an accelerating voltage of 5 kV in high vacuum (10^−6^ mbar). 

The zeta potential test was performed to analyze the potential variation of the GO nanosheets after PA modification by using the Zeta sizer 3000 instrument (Malvern Panalytical, Malvern, UK.

The coating surface investigation were performed by electron microscopy using a Zeiss Sigma VP (Zeiss, Oberkochen, Germany) field emission scanning electron microscope (FE-SEM), endowed with a Bruker Quantax energy dispersive X-ray (EDX) spectrometry microanalysis detector (Bruker, Billerica, MA, USA), which was used to investigate the surface morphologies and element content of different covered GO-PA panels. 

Optical analysis of the samples was obtained by using a Hirox digital microscope KH-8700 (Hirox, Tokyo, Japan), with optical microscope in xyz (3D) and mapping mode. Further, MX(G)-5040Z lens was used to record the optical images.

With the portable and compact roughness tester, Surftest SJ-210, series 178 (Mitutoyo S.r.l., Milan, Italy), the surface roughness (Ra) was calculated by the following, Equation (2),

(2) $$\mathrm{Ra}=\frac{1}{N}\overset{n}{\underset{i=1}{\sum }}\left|\mathrm{Yi}\right|$$

where Ra represents the arithmetic mean of the absolute values of the deviations of the evaluation profile (Yi) from the mean line. The JIS2001 roughness standard was employed for the measurement conditions, five sampling lengths, lengths of cut-off (λs = 2.5 mm, λc = 0.8 mm), and a stylus translation speed of 0.5 mm/sec. The average profile was obtained on n. 4 roughness profiles per type of sample.

The pull-off adhesion test was performed by a Lloyd LR10K (AMETEK GmbH, Meerbusch, Germany) universal testing machine, according to the ASTM D4541 standard test method for pull-off strength of coatings. During the test, steel metal dolls were attached perpendicularly on the steel or aluminum metal samples, uncoated and coated with GO-PA/APTES or GO-PA/GPTMS. The test conditions were load cell 10 KN, with pre-load 1.00 N and speed 1 mm/min. 

A commercial cross-hatch adhesion tester (SAMA Tools SADT502-5, SAMA Italia, Viareggio, Italy) was employed for the testing of the adherence of coating films to metallic substrates, according to ASTM D3359e2 standard test method for measuring adhesion by tape. An approximately 10 × 10 cm grid incision was made in a selected test area using an appropriate cutter, characterized by horizontally and vertically spaced (2 mm) incisions on the surface. By a soft brush, the excess particles produced in the region were removed. Moreover, a 3 M adhesive tape was applied on the cutting grid by a light pressure and then peeled off in an orderly motion. The ISO 2409:2013 reference images were used for comparison with the selected pictures, in order to visually evaluate the condition of the damage. Based on the number of flaked off squares and appearance, a cross-cut parameter, ranging from 0 (very good adhesive strength) to 5 (extremely poor adhesive strength), was assigned.

A high precision digital coating thickness gauge, SAMA TOOLS–SA8850 (S.A.M.A Italia S.r.l., Viareggio, Italy), was used to measure the thickness of the films on metal bases in a non-destructive way. A grid was created on the metal specimen to acquire, at the intersection points, the different thickness values for the entire sample.

A PalmSens2 potentiostat from PalmSens (Houten, The Netherlands) was used for the linear polarization measurements. In particular, the analyses were conducted in three electrodes cell, using NaCl 0.1 M solution (Ag/AgCl) (KCl 3M) reference electrode and Pt counter electrode, working electrode as the tested surfaces, with 1.77 cm^2^ surface area. The linear polarization was performed by measuring open circuit potential (EOC) for 10 s and scanning potential from EOC ± 0.2 V, with 1 mV potential steps, 1.7 mV/s as scan rate, and the data analyzed by PSTrace software, in order to determine the Tafel slope of the polarization in the corrosion mode.

## 4. Conclusions

This work presents the efficient design and development of polymeric silane-based, multicomponent, water-based, and eco-friendly coatings, featuring cross-linked functionalized GO with PA nanofillers, as obtained by sol–gel technology. The synthetized nanostructured coatings were tested as an anticorrosive finishing for a metal surface. It has been demonstrated that the functionalization of GO nanosheets with PA is a key step for enhancing their dispersion in polymeric coatings. The results of the FT-IR spectroscopy, UV–Vis analysis, and SEM indicated the successful decoration of PA molecules on the surface of GO. Anticorrosive performance was tested by polarization measurements and neutral salt spray test, thus confirming that GO-PA nanofillers enhance the barrier and corrosion protection properties of the silane-based coatings. This behavior is attributable to the coatings’ enhanced impermeability and adhesive power, as a result of the addition of uniformly dispersed GO-PA nanofillers. In this context, phytic acid is a type of natural organic coordination molecule that, because of its special properties, such as its non-toxicity, biocompatibility, and environmental friendliness, can serve as a safe and green inhibitor of corrosion. The developed functional waterborne multicomponent anticorrosive coatings are expected to pave the way for other efficient cross-linked coatings that may be used as a primer between metal surfaces and a top aesthetic paint, and they are able to: (i) improve the performances and lifetime of the painted metal surfaces against corrosion, due to chemical and environmental agents, (ii) reduce the maintenance phases of the metal surfaces, and (iii) lead to an overall great economic advantage for different metal-based application fields. Therefore, the waterborne, sol–gel-based hybrid coatings bearing GO intercalated PA anticorrosive agents, as developed in this study, could represent a useful, environmentally safe, and easy method of achieving good anti-corrosive and protective coatings for various metal-based application fields.

## Figures and Tables

**Table 1:** Recent sol–gel-based functional coatings for anticorrosion treatments of different surfaces.

Functional Sol	Anticorrosion Agent	Treated Surface	Ref.
γ-glycidoxypropyltri-methoxysilane, tetraethoxysilane, methyltriethoxysilane	NaX zeolite crystals hosting Zn^2+^ and mercaptobenzimidazole	Carbon steel	[69]
Titanium (IV) butoxide	AgNP/PTFE	316L Stainless steel	[70]
Tetraethylorthosilicate	ZrO_2_	316L Stainless steel	[71]
Tetraethoxysilane, methyltrimethoxysilane	Silicate, borosilicate and copper-doped borosilicate	AISI 316 L Stainless steel	[72]
Tetraethoxysilane, glycidoxypropyltrimethoxysilane	Cerium modified montmorillonite	Aluminum alloy AA2024	[73]
Tetraethoxysilane, glycidoxypropyltrimethoxysilane	PEO/sodium montmorillonite	Aluminum alloy AA2024	[74]
Tetraethylorthosilicate, TEOS, γ-glycidyloxypropyltrimethoxysilane	MIL-53 (Al) nanoparticles	Aluminum alloy AA2024	[75]
Tetraethylorthosilicate, methyltriethoxysilane	l-Glutamine, l-methionine, l-aspartic acid, and l-alanine	ZE41 magnesium alloy	[76]
Tetraethyl orthosilicate, (3-glycidyloxypropyl) trimethoxysilane	Aminated and sodium dodecyl sulfate-stabilized fullerene nanoparticles	AM60B magnesium alloy	[77]
Tetraethoxysilane, 3-glycidoxypropyl trimethoxysilane	Hydroxylated nanodiamond	AM60B magnesium alloy	[78]
(3-aminopropyl)triethoxysilane, (3-Glycidyloxypropyl)trimethoxysilane	Graphene oxide intercalated phytic acid	AQ-36 aluminum and QD-36 carbon steel	This work

**Table 2:** Roughness profile values of the different coatings.

Sample Code	Ra (μm)	Rz (μm)	Sample Code	Ra (μm)	Rz (μm)
Al(AQ-36)	4.64	11.75	St (QD-36)	5.84	14.05
Al + GO-PA/APTES	9.23	20.45	St + GO-PA/APTES	5.09	12.55
Al + GO-PA/GPTMS	4.45	11.20	St + GO-PA/GPTMS	5.48	12.73

**Table 3:** Thickness values of the uncoated and coated samples obtained from the chromatic maps.

Sample Code	T (μm)	Sample Code	T (μm)
Al(AQ-36)	102	St (QD-36)	60
Al + GO-PA/APTES	102	St + GO-PA/APTES	62.5
Al + GO-PA/GPTMS	160	St + GO-PA/GPTMS	80

**Table 4:** Calculation parameters for AQ-36 aluminum samples.

Name	E_corr_ (V)	i_corr_ (A/cm^2^)	R_p_ (Ω)	PE (%)	CR (mm/Year)
Al (AQ-36)	−1.022	4.29 × 10^−7^	1.87 × 10^5^	0	5.0 × 10^−3^
Al + GO-PA/GPTMS	−0.756	1.88 × 10^−8^	1.51 × 10^6^	95.62	2.05 × 10^−4^
Al + GO-PA/APTES	−0.834	4.44 × 10^−9^	2.88 × 10^6^	98.97	4.84 × 10^−5^

**Table 5:** Calculation parameters for QD-36 steel samples.

Name	E_corr_ (V)	i_corr_ (A/cm^2^)	R_p_ (Ω)	PE (%)	CR (mm/Year)
St (QD-36)	−0.309	3.02 × 10^−5^	893.1	0	0.351
St + GO-PA/GPTMS	−0.281	1.68 × 10^−5^	1372	41.06	0.196
St + GO-PA/APTES	−0.253	1.63 × 10^−5^	1563	46.03	0.190

**Table 6:** Q-PANEL standard test substrate dimensions.

Panel Type	Stock Number	Size W × L (mm)	Thickness (mm)
Type AQ	AQ-36	76 × 152	0.81
Type QD	QD-36	76 × 152	0.81

## Data Availability

Not applicable.
